# Nationwide Deployment of a Serious Game Designed to Improve COVID-19 Infection Prevention Practices in Switzerland: Prospective Web-Based Study

**DOI:** 10.2196/33003

**Published:** 2021-11-25

**Authors:** Melanie Suppan, Loric Stuby, Stephan Harbarth, Christophe A Fehlmann, Sophia Achab, Mohamed Abbas, Laurent Suppan

**Affiliations:** 1 Division of Anesthesiology Department of Anesthesiology, Clinical Pharmacology, Intensive Care, and Emergency Medicine University of Geneva Hospitals and Faculty of Medicine Geneva Switzerland; 2 Genève TEAM Ambulances Geneva Switzerland; 3 Infection Control Programme and WHO Collaborating Centre on Patient Safety University of Geneva Hospitals and Faculty of Medicine Geneva Switzerland; 4 Division of Emergency Medicine Department of Anesthesiology, Clinical Pharmacology, Intensive Care, and Emergency Medicine University of Geneva Hospitals and Faculty of Medicine Geneva Switzerland; 5 Specialized Facility in Behavioral Addictions ReConnecte Geneva University Hospitals Geneva Switzerland; 6 WHO Collaborating Center in Training and Research in Mental Health University of Geneva Geneva Switzerland

**Keywords:** COVID-19, serious game, infection prevention, SARS-CoV-2, prospective, web-based, deployment, prevention, gaming, public health, dissemination, health information, behavior, survey

## Abstract

**Background:**

Lassitude and a rather high degree of mistrust toward the authorities can make regular or overly constraining COVID-19 infection prevention and control campaigns inefficient and even counterproductive. Serious games provide an original, engaging, and potentially effective way of disseminating COVID-19 infection prevention and control guidelines. *Escape COVID-19* is a serious game for teaching COVID-19 infection prevention and control practices that has previously been validated in a population of nursing home personnel.

**Objective:**

We aimed to identify factors learned from playing the serious game Escape COVID-19 that facilitate or impede intentions of changing infection prevention and control behavior in a large and heterogeneous Swiss population.

**Methods:**

This fully automated, prospective web-based study, compliant with the Checklist for Reporting Results of Internet E-Surveys (CHERRIES), was conducted in all 3 main language regions of Switzerland. After creating an account on the platform, participants were asked to complete a short demographic questionnaire before accessing the serious game. The only incentive given to the potential participants was a course completion certificate, which participants obtained after completing the postgame questionnaire. The primary outcome was the proportion of participants who reported that they were willing to change their infection prevention and control behavior. Secondary outcomes were the infection prevention and control areas affected by this willingness and the presumed evolution in the use of specific personal protective equipment items. The elements associated with intention to change infection prevention and control behavior, or lack thereof, were also assessed. Other secondary outcomes were the subjective perceptions regarding length, difficulty, meaningfulness, and usefulness of the serious game; impression of engagement and boredom while playing the serious game; and willingness to recommend its use to friends or colleagues.

**Results:**

From March 9 to June 9, 2021, a total of 3227 accounts were created on the platform, and 1104 participants (34.2%) completed the postgame questionnaire. Of the 1104 respondents, 509 respondents (46.1%) answered that they intended to change their infection prevention and control behavior after playing the game. Among the respondents who answered that they did not intend to change their behavior, 86.1% (512/595) answered that they already apply these guidelines. Participants who followed the German version were less likely to intend to change their infection prevention and control behavior (odds ratio [OR] 0.48, 95% CI 0.24-0.96; *P*=.04) and found the game less engaging (*P*<.001). Conversely, participants aged 53 years or older had stronger intentions of changing infection prevention and control behavior (OR 2.07, 95% CI 1.44-2.97; *P*<.001).

**Conclusions:**

Escape COVID-19 is a useful tool to enhance correct infection prevention and control measures on a national scale, even after 2 COVID-19 pandemic waves; however, the serious game's impact was affected by language, age category, and previous educational training, and the game should be adapted to enhance its impact on specific populations.

## Introduction

### Background and Importance

Vaccination campaigns against SARS-CoV-2 have been gathering momentum, but most countries remain months away from reaching group immunity [1-4], provided that such immunity can be attained [5,6]. The almost incessant emergence of new variants is concerning [7]—all the more so as some variants have been shown to evade the immune response acquired by vaccination or by prior infection [8,9]. Viral dissemination may even be facilitated, if normal social interactions are restored and social distancing measures are eliminated, as countries face ever increasing social and economic pressures [10-12]. Moreover, many people experience a rather intense state of tiredness [13-15] coupled with a rather high degree of mistrust toward public health authorities [2,16-18], which can result in a rapidly progressive slackening of infection prevention and control procedures [19-21] and which may even affect health care workers [22]. Switzerland is no exception, and conspiracy theories also thrive in this country [23]. Strengthening infection prevention and control messages and promoting adequate behavior is, therefore, more important than ever; however, regular or aggressive information campaigns might prove counterproductive [21]. These latter strategies were intensively used during the early waves of the pandemic; however, new methods of communication should be considered to increase adherence to infection prevention and control guidelines.

To disseminate COVID-19 prevention messages in an original and engaging way, a transdepartmental and multidisciplinary development team created Escape COVID-19 [24], a serious game based on Nicholson’s concept for meaningful gamification [25]. By increasing the players’ engagement, serious games, which also enhance knowledge, satisfaction, and skills [26], can help promote adequate behaviors [27,28]. The impact of Escape COVID-19 was assessed in a population of nursing home employees [29]: In a triple-blind controlled trial, the self-reported likelihood of changing infection prevention and control practices was almost 4 times higher in the group of participants who had followed the serious game [29]. However, Escape COVID-19 was only available in French at the time of this prior study, and the population in which it was tested did not allow us to determine whether socioeconomic status or cultural differences influenced its uptake. We hypothesized that receptiveness to messages promoting COVID-19 infection prevention and control behaviors might depend on socioeconomic status and cultural specificities [30].

### Objective

The objective of this study was to identify factors learned from playing Escape COVID-19 that favor or impede intentions of changing infection prevention and control behavior in a larger and more heterogeneous population than that used in the previous study [29].

## Methods

### The Escape COVID-19 Serious Game

The development and features of the Escape COVID-19 serious game have been described previously [24,29]. Briefly, this game was created using Storyline 3 (Articulate Global LLC) and developed using the SERES framework [31], an iterative development approach, to ensure that scientific and design foundations were evidence-based and adapted to the target audience. The different types of players described by Bartle [32] were taken into consideration during the development of Escape COVID-19; therefore, some graphics, game mechanics, and narratives were developed to target achievers, explorers, and socializers.

There is little consensus on what actually defines a serious game; however, according to the conceptual framework created by Tan and Zary [33], Escape COVID-19 fits the definition of a serious game. This framework [33], which describes the criteria required to determine whether a specific material is indeed a serious game, comprises 3 clusters: user experience, play, and learning. Each of these clusters includes 6 base markers, and a minimum of 4 markers per cluster is required to declare that the material under consideration is indeed a serious game. Escape COVID-19 complies with all 18 markers.

**Figure 1:**
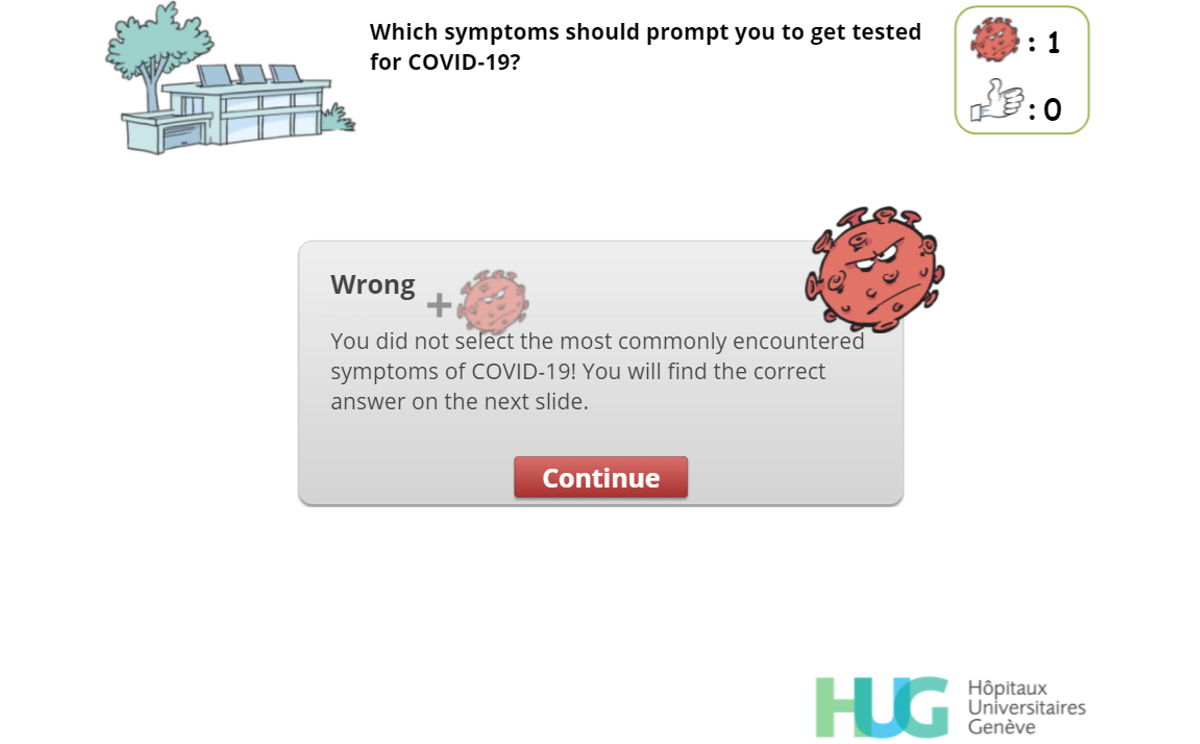
Screenshot showing an example—the player has given a wrong answer, and their viral count rose accordingly.

Escape COVID-19 contains 4 different levels and takes approximately 15 minutes to complete. Each level represents settings that health care workers typically encounter during a work day (at home, on the road, in communal areas, and in the ward). Players are faced with meaningful choices and are consistently provided with customized, relevant feedback [34]. Adequate infection prevention and control behaviors are rewarded (by an increase in their “thumbs-up” count), while dangerous behaviors lead to an increase in the viral count (Figure 1).

**Figure 2:**
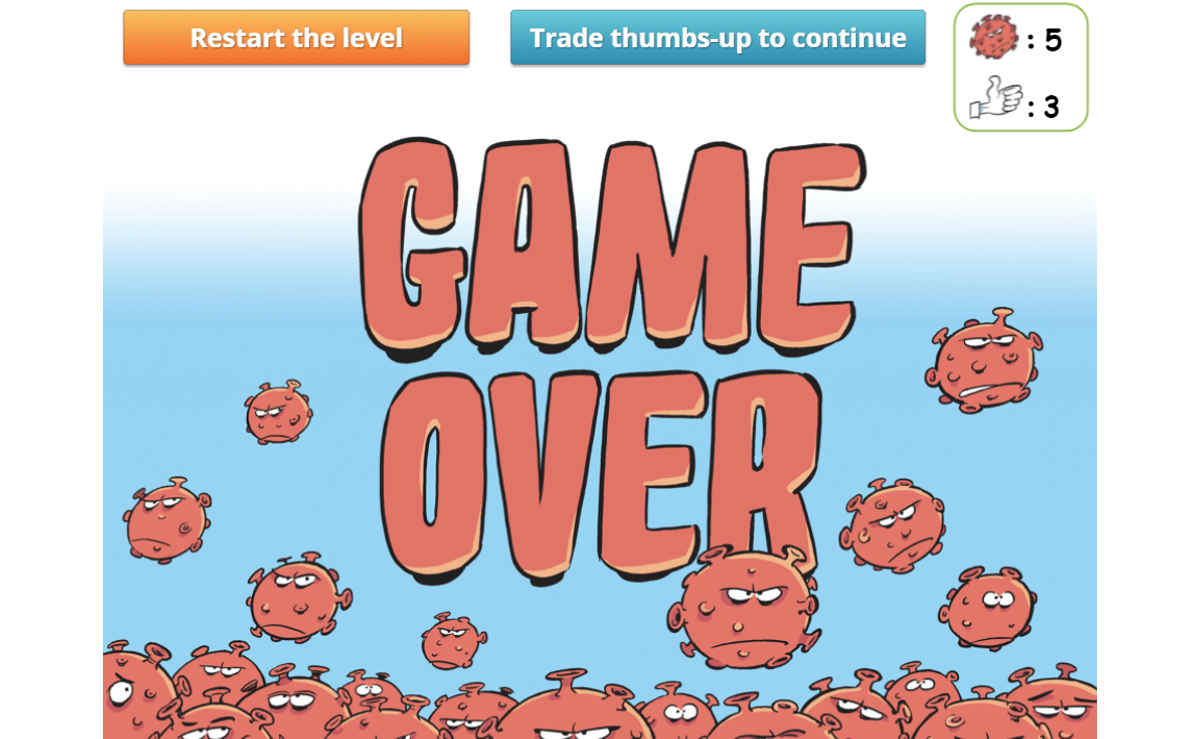
Game over screenshot.

If the virus count reaches a value of 5, a game over screen is displayed (Figure 2). The player can then choose to restart the level or to exchange their thumbs-up count for an equivalent reduction in virus count.

**Figure 3:**
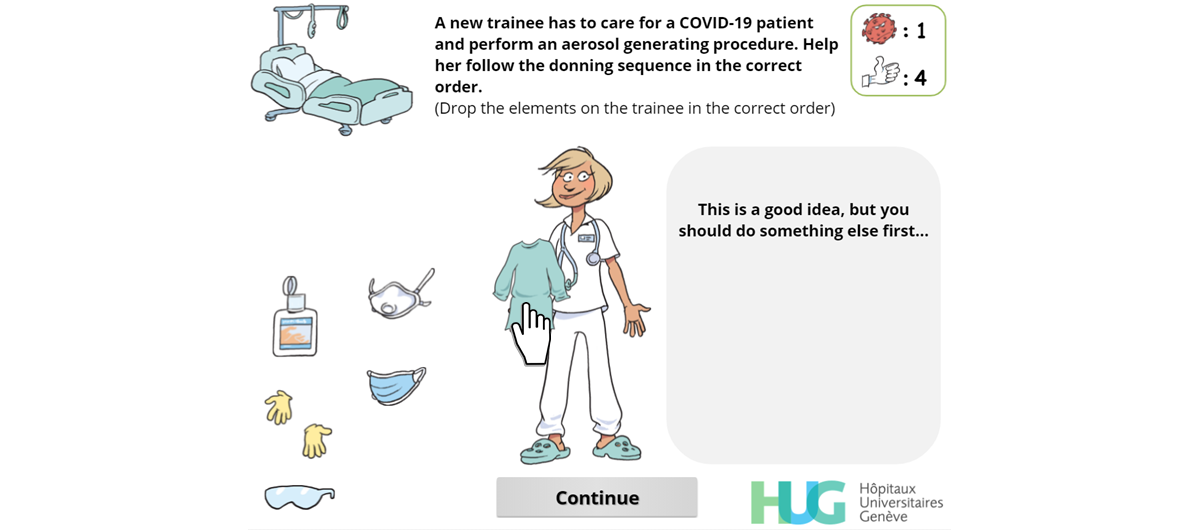
In the donning sequence interaction, the player is asked to drag and drop the appropriate personal protective equipment items in the correct order, and live feedback is displayed in the grey area on the right.

An abridged version, in which the fourth and hardest level (the ward—Figure 3) was not available to players, was also developed (at the request of the Swiss National Science Foundation).

### Study Design, Setting, and Participants

**Figure 4:**
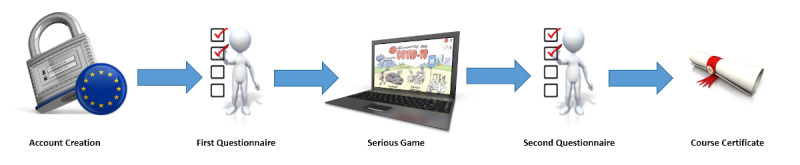
Study design. The European Union flag (on the left at the account creation step) signifies that account and data management were in compliance with the General Data Protection Regulation.

We conducted a web-based, fully-automated prospective cohort study in accordance with the Checklist for Reporting Results of Internet E-Surveys (Figure 4) [35]. A declaration of *no objection* was issued by the regional research ethics committee (Req-2021-00600) as this project did not fall within the scope of the Swiss Act on Research involving Human Beings [36]. A disclaimer containing a data policy statement was displayed on the registration form. Data were collected between March and June 2021.

Switzerland is a federal country composed of 26 cantons. There are 4 official languages in Switzerland: German and Swiss German, which are usually grouped as one (62.1%), French (22.8%), Italian (8.0%), and Romansh (0.5%). English is the main language for 5.7% of the permanent resident population [37]. We developed the original version of the Escape COVID-19 serious game in French [24], which we then translated into English. Translations in German and Italian were requested and paid for by the Swiss National Science Foundation (National Research Program 78 framework [38]). A separate translation in Romansh was not requested because Romansh-speaking people in Switzerland generally also speak either German or Italian.

While health care workers were the primary target of this serious game [24], because many of its infection prevention and control messages also apply, we included non–health care workers in this study.

To recruit participants, the Swiss National Science Foundation financed the dissemination of the link to the Escape COVID-19 website [39]. Public and private health care organizations in Switzerland were contacted, and managers were asked to spread the link by different means (such as by email and newsletters). Ten organizations, including the Swiss Red Cross and the Swiss Society for Public Health, decided to endorse the game and allow their logo to be displayed on the website and on the course completion certificate. To enhance participation even further, the Geneva University Hospitals published a press release [40], which was relayed by *Agence France-Presse* [41].

### Web Platform and Study Sequence

**Figure 5:**
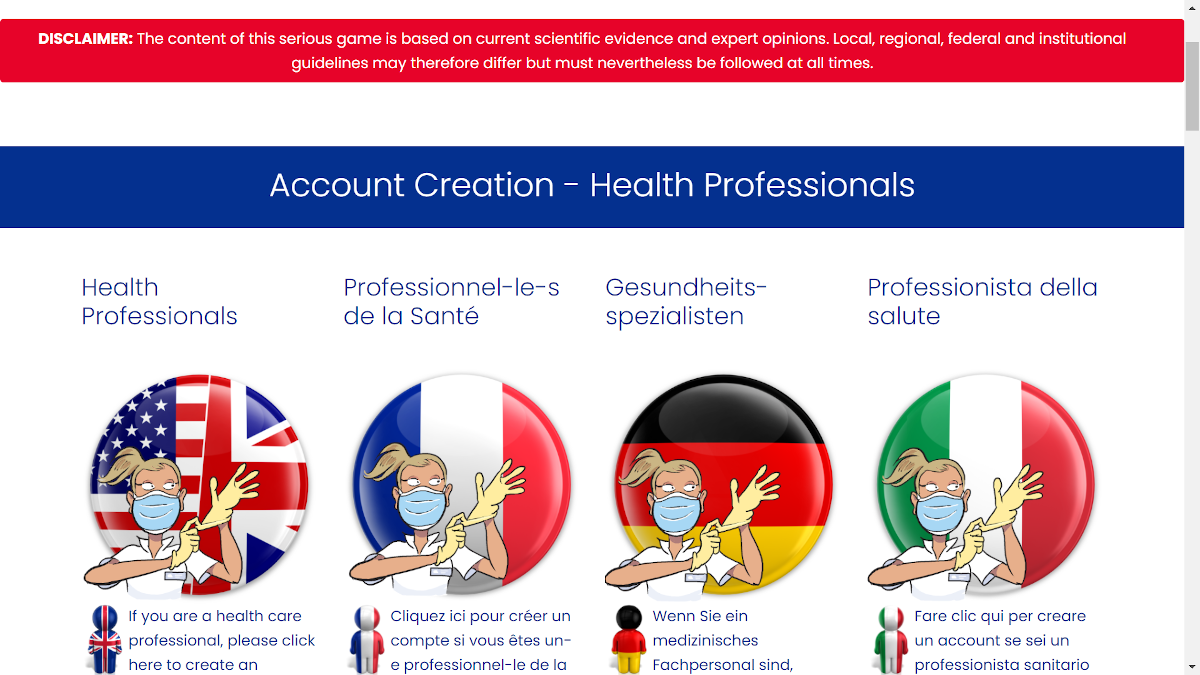
Front page of the website.

A multilingual website [39] was developed using a content management system (Joomla!, version 3.9; Open Source Matters Inc). The front page displayed a disclaimer to address concerns regarding potentially contradicting guidelines immediately above the language-specific links used for account creation (Figure 5). Dedicated links were available to allow non–health care workers to create specific accounts.

**Figure 6:**
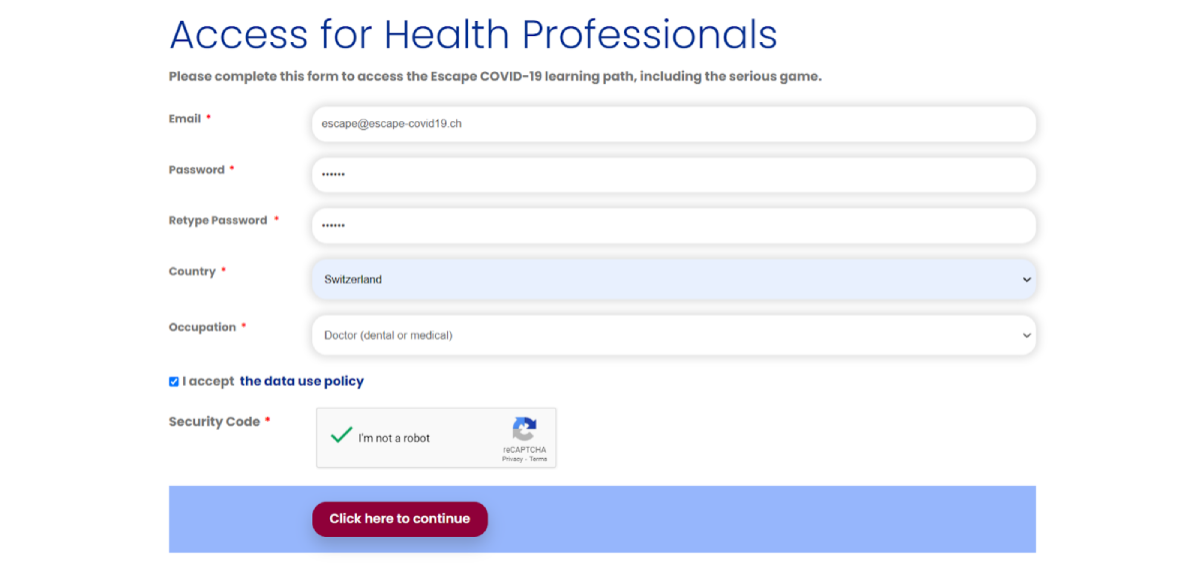
Registration form example.

Participants registered using Joom Donation MembershipPro (version 2.1; Joomla Extensions by JoomDonation), which allows an extensive customization of the registration fields and the addition of specific fields. For instance, the occupation field differed for those who were health care workers and those who were not health care workers. In addition, respondents who were not health care workers were asked to select the highest degree or level of school that they had completed. All fields, including CAPTCHA (Completely Automated Public Turing test to tell Computers and Humans Apart) and a checkbox to accept the data use policy, were required before participants were automatically logged in (Figure 6).

Participants were immediately redirected to a demographic questionnaire (gender, age, and COVID status). All questionnaires were created using Community Surveys (version 5.5; Bulasikku Technologies Pvt Ltd), which allows for the use of branching logic. Regular expressions were used to avoid invalid entries. Respondents who were not health care workers were asked whether they wanted to follow the full or abridged version of the Escape COVID-19 serious game.

After completing the demographic questionnaire, participants were able to launch and play Escape COVID-19. After completing the game, a second questionnaire was displayed (Table 1).

**Table 1:** Second questionnaire.

Questions, response options, and response-dependent questions			
**After playing this serious game, are you going to change any of your infection prevention practices?**			
	**Yes**		
		**What areas will these changes affect?^a^**	
			Not going to work if you have symptoms compatible with COVID-19
			Protecting yourself from both your colleagues and your patients^b^
			The donning sequence^c^ when dealing with procedures CARRYING a risk of aerosolization^b^
			The donning sequence when dealing with procedures NOT CARRYING a risk of aerosolization^b^
			Changing nonsterile gloves more frequently^b^
			Practicing hand hygiene more frequently
			Disinfecting your workplace
			Handling the face mask more carefully
			Protecting yourself from asymptomatic people as well as from symptomatic ones
		**You are now going to use:^d^**	
			Face masks
			N95 respirator masks
			Eye protection^b^
			Nonsterile gloves^b^
		**Which of these elements greatly contributed to your intention to modify your practices?^e^**	
			The information given in the serious game
			The feeling of playing an important role in the common effort against the epidemic
			The probability of infecting a relative
			One should follow the procedures
			Another reason^f^
	**No**		
		**Why will your practices not change?^d^**	
			I already apply all these guidelines
			The information given in this serious game does not apply to my situation
			The information given in the serious game was not helpful
			I do not believe these measures to be useful
			I disagree with these measures
			Another reason^f^
		**What could have motivated you to change your practices?^e^**	
			Better understand the reasons behind the recommendations
			A greater probability of infecting a relative
			The feeling of having an important role in the common effort against the epidemic
			Nothing—I could not have been convinced by any argument
			Other^f^
Difficulty of the serious game^g^			
**Please rate the following items from 1 (strongly disagree) to 5 (strongly agree):**			
	This serious game is engaging		
	This serious game is meaningful		
	This serious game is useful		
	This serious game is boring		
	I will recommend this serious game to my friends and relatives		
	I will recommend this serious game to my colleagues		
Duration of the serious game^h^			
Do you have any further comments to make regarding this serious game?			

^a^Response options were in the form of a 5-point Likert scale from 1 (not at all) to 5 (very much).

^b^This option was not shown to non–health care workers who had elected to follow the abridged version of the serious game.

^c^The *donning sequence* refers to the order in which the personal protective equipment items should be put on.

^d^Response options were in the form of a 5-point Likert scale from 1 (much less) to 5 (much more).

^e^Multiple choice question (more than one possible answer).

^f^A free-text field was displayed when this option was selected.

^g^Response options were in the form of a 5-point Likert scale from 1 (too easy) to 5 (too difficult).

^h^Response options were in the form of a 5-point Likert scale from 1 (much too short) to 5 (much too long).

Participants were unable to bypass any step of the study path by virtue of the Access Control List feature in Joomla!. Sourcerer (version 8, Regular Labs) was used to embed the appropriate PHP (Hypertext Preprocessor) functions (JUserHelper::removeUserFromGroup and JUserHelper::addUserToGroup) to ensure that participants were redirected to the appropriate step when resuming the study path. The Conditional Content component (version 3, Regular Labs) was used to display text and links allowing participants to easily resume the study path.

### Data Collection

All data were automatically stored on an encrypted MySQL-compatible database (MariaDB, version 5.5.5; MariaDB Corporation Ab) located on a Swiss server. Participants were able to access and delete their accounts and data at any time. Data from all the participants who created an account on the study platform between March 9 and June 9, 2021 were included regardless of their professional status. Participants were allowed to delete their accounts after playing the game, in which case all data associated with their account were automatically removed from the database and could, therefore, be neither retrieved nor included in analysis.

Given the design of this study, there was no predetermined sample size, and no sampling scheme was used. Therefore, the participants who did not delete their accounts represented a convenience sample.

### Outcomes

The primary outcome was the proportion of participants reporting that they were willing to change their infection prevention and control behavior (ie, by answering “Yes” to the first question of the second questionnaire). Some secondary outcomes depended on the participants’ willingness to change infection prevention and control behavior: In participants willing to change behavior, secondary outcomes were the infection prevention and control areas affected and the intensity of the participants’ willingness to change. The presumed evolution in the use of specific personal protective equipment items was also assessed, as were the elements motivating this willingness to change behavior. In participants unwilling to change infection prevention and control behavior, secondary outcomes were assessment of the reasons for refusing to change and of the potential motivators which could have induced a willingness to change. Other secondary outcomes such as length, difficulty, and willingness to recommend the serious game to either friends or colleagues were assessed regardless of the participants’ intention to change behavior. Perceived meaningfulness and usefulness of the game and impression of engagement and boredom while playing the serious game were also assessed in the whole sample.

### Data Curation and Statistical Analysis

Data were extracted from the database and imported into Stata (version 15; StataCorp LLC) for data curation. Records of participants who did not complete the first questionnaire were excluded. The curated DTA file is available in Multimedia Appendix 1. Descriptive statistics (frequency, relative percentage, mean, and standard deviation) were used when appropriate. As the serious game has already proven its usefulness in health care workers [29], the primary outcome, intention of changing infection prevention and control behavior, was first analyzed using univariate logistic regression according to professional status (health care workers versus non–health care workers). Multivariable logistic regression was then performed to further adjust for gender, COVID status, language, and age. The assumption of log-linearity of age was assessed graphically. As age was not log-linear, it was categorized according to its quartiles. Goodness of fit was checked using the Hosmer-Lemeshow test. Among non–health care workers, univariate and multivariable logistic regression were performed to determine whether language or other specific factors such as gender, age category, occupation, level of education, or playing the abridged version of the serious game rather than the full game were associated with answering the second questionnaire after completing the serious game. The actual values, from 1 (not at all) to 5 (very much) based on Likert scales, were first used to compute the secondary outcomes. No weighting was applied. For these outcomes, inconsistent answers (ie, those from participants who gave either the minimum or maximum rating for both boredom and engagement) were excluded.

The likelihood ratio test was used to determine whether the parallel lines or proportional odds assumption was met. When met, multivariable ordered logistic regression was used to search for an association between participants’ characteristics and secondary outcomes related to the perception of the serious game (engagement and meaningfulness). Because the aforementioned assumption was not met, 2 generalized ordered logistic models were generated using 2 different secondary outcomes as dependent variables: boredom and usefulness. Willingness to change, language, gender, age category, COVID status, occupation, and level of education were used as predictive variables in these models.

## Results

**Figure 7:**
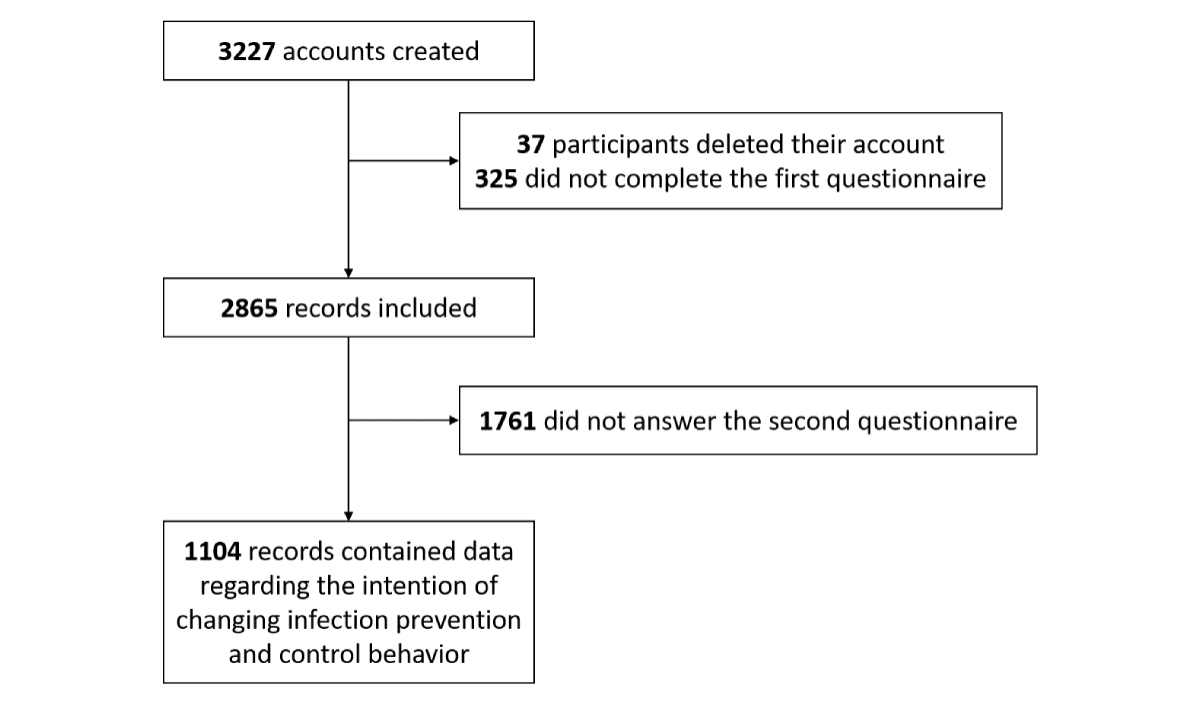
Study flowchart.

From March 9 to June 9, 2021, a total of 3227 accounts were created on the platform: 37 participants chose to delete their accounts, and 325 were excluded as they did not complete the first questionnaire (Figure 7). The characteristics of the 2865 participants included in the analysis are described in Tables 2 and 3.

Most health care workers were nurses (53.0%, 967/1823). Physicians accounted for 11.9% of participants (216/1823), and nursing assistants accounted for 8.1% (148/1823). Other health care professions accounted for 27.0% of the health care worker population.

**Table 2:** Participant characteristics.

Characteristic		Non–health care worker (n=1042)	Health care worker (n=1823)
**Language, n (%)^a^**			
	English	86 (8.3)	83 (4.6)
	French	515 (49.4)	646 (35.4)
	German	415 (39.8)	1064 (58.4)
	Italian	26 (2.5)	30 (1.7)
**Gender, n (%)**			
	Male	395 (37.9)	404 (22.2)
	Female	630 (60.5)	1404 (77.0)
	Other	17 (1.6)	13 (0.7)
	Missing	0 (0)	2 (0.1)
Age, mean (SD)		40.9 (13.8)	41.6 (13.6)^b^
**COVID status, n (%)^a^**			
	Negative or not tested	905 (86.9)	1447 (79.4)
	Positive or isolated	3 (0.3)	6 (0.3)
	Cured	84 (8.1)	232 (12.7)
	Refused to answer	50 (4.8)	136 (7.5)
	Missing	0 (0)	2 (0.1)

^a^Percentage totals may exceed 100% due to rounding.

^b^n=2 values were missing.

**Table 3:** Additional characteristics of non–health care workers.

Characteristic		Non–health care workers, n (%)
**Occupation**		
	Health or social	251 (24.1)
	Business	49 (4.7)
	Hospitality	20 (1.9)
	Manufacturing	35 (3.4)
	Public sector or education	252 (24.2)
	Transport or retail	28 (2.7)
	Other	335 (32.1)
	Missing	72 (6.9)
**Degree of education**		
	Mandatory school	49 (4.7)
	Secondary education	39 (3.7)
	Professional diploma	289 (27.7)
	High school graduate	149 (14.3)
	University graduate	430 (41.3)
	Other	60 (5.8)
	Missing	26 (2.5)
**Version played**		
	Abridged	576 (55.3)
	Full	466 (44.7)

Of the 2865 participants included in the analysis, 1104 (38.5%) provided information regarding their intentions of changing infection prevention and control behavior, and 1061 fully completed the second questionnaire. Of those, 477 (45.0%) generated a course completion certificate.

Among non–health care workers, the only factor associated with the probability of answering the second questionnaire was having completed the abridged version, which was significant after adjusting for playing the abridged version rather than the full one and for gender, age, occupation, and level of education (odds ratio [OR] 1.96, 95% CI 1.48-2.59). In health care workers, an association between language and the probability of answering the questionnaire was present after univariate analysis (*P*<.001) and remained after adjusting for gender, age category, profession, and COVID status (*P*=.002). In this group, participants who refused to detail their COVID status were less likely to answer this questionnaire (OR 0.49, 95% CI 0.32-0.75).

Among participants who answered the second questionnaire, less than half (509/1104, 46.1%) intended to change their infection prevention and control behavior after playing the game. However, among those who did not intend to change their infection prevention and control behavior, the vast majority answered that they already apply these guidelines (Table 4). Few participants (13/595, 2.2%) answered that they disagreed with these measures or that they did not believe such measures to be useful.

Participants intending to change infection prevention and control behavior were generally radical in their willingness to change certain aspects of the practices (not at all: 1003/4085 answers, 24.6%; very much: 1795/4085 answers, 43.9%).

Most of them were not likely to change their use of specific personal protective equipment items, the only exception being related to the use of protective goggles (156/383, 40.7% answered that they would use goggles more or much more after playing the serious game).

Participants who answered that they intended to change their infection prevention and control behavior were mostly motivated by the information contained in the serious game and by the feeling of playing an important role in the common effort against the epidemic (Table 5).

**Table 4:** Reasons given for not changing infection prevention and control behavior.

Reason	Non–health care worker (n=309), n (%)^a^	Health care worker (n=286), n (%)^a^
Already applies these guidelines	288 (93.2)	224 (78.3)
The information given in the serious game did not apply to the participant’s situation	33 (10.7)	78 (27.3)
Disagrees with these measures	3 (1.0)	10 (3.5)
The information given in the serious game was not considered helpful	5 (1.6)	2 (0.7)
Did not believe these measures to be useful	2 (0.6)	2 (0.7)
Another reason	6 (1.9)	19 (6.6)

^a^Multiple responses are possible; therefore, percentages do not add to 100%.

**Table 5:** Reasons associated with the willingness of changing infection prevention and control behavior.

Reason	Non–health care worker (n=124), n (%)^a^	Health care worker (n=385), n (%)^a^
The information given in the serious game	70 (56.5)	208 (54.0)
The feeling of playing an important role in the common effort against the epidemic	76 (61.3)	200 (51.9)
One should follow the procedures	43 (34.7)	144 (37.4)
The probability of infecting a relative	57 (46.0)	129 (33.5)
Another reason	3 (2.4)	8 (2.1)

^a^Multiple responses are possible; therefore, percentages do not add to 100%.

Univariate logistic regression showed that, compared to non–health care workers, health care workers were more likely to intend to change their infection prevention and control behavior (OR 3.35, 95% CI 2.59-4.34). This association was reinforced after adjusting for age category, gender, language, and COVID status (OR 3.88, 95% CI 2.94-5.13). The 2 elements with significant associations were German language (OR 0.48, 95% CI 0.24-0.96) compared to reference category (English) and being aged 53 years or older (OR 2.07, 95% CI 1.44-2.97) compared to reference category (11-30 years).

Among non–health care workers, belonging to the highest age category (53 years or older: OR 3.33, 95% CI 1.66-6.68) and playing the full rather than the abridged version of the serious game (OR 4.45, 95% CI 2.63-7.55) were associated with a more significant willingness to change infection prevention and control behavior. There was no significant difference in this outcome related to either occupation (*P*=.42) or educational status (*P*=.11).

Thirty-three records (33/1061, 3.1%) were excluded because they contained either minimal or maximal ratings for both the engagement and boredom items. The serious game was generally considered engaging (mean 3.9, SD 1.0), meaningful (mean 4.4, SD 0.9), and useful (mean 4.3, SD 0.9). Boredom was rated rather low (mean 1.9, SD 1.1).

Perceived meaningfulness was affected by 4 different factors. Non–health care workers who intended to change their infection prevention and control behavior were more likely to find the game meaningful (OR 2.40, 95% CI 1.44-3.99). Those who had chosen to play the full version of the game were also more likely to find the game meaningful (OR 1.74, 95% CI 1.05-2.88). Participants who did not identify themselves as belonging to the male or female gender found the game to be less meaningful (OR 0.14, 95% CI 0.03-0.68).

Likewise, non–health care workers who intended to change their infection prevention and control behavior, and those who had chosen to play the full version of the game were more likely to find it engaging (OR 2.73, 95% CI 1.71-4.33 and OR 2.24 95% CI 1.39-3.60, respectively). Non–health care workers who identified themselves as belonging to the female gender also found the game more engaging (OR 1.58, 95% CI 1.02-2.45), while participants who did not identify themselves as either male or female found it less engaging (OR 0.19, 95% CI 0.05-0.76). Participants who played the German version of the game also found it less engaging (OR 0.15, 95% CI 0.06-0.40).

Health care workers were more likely than non–health care workers to recommend this serious game to their colleagues (OR 1.87, 95% CI 1.49-2.35). They were also more likely to recommend it to their relatives than non–health care workers were (OR 1.43, 95% CI 1.14-1.79). Regarding difficulty, Escape COVID-19 was considered to be well balanced by 71.3% (756/1061), and more health care workers (534/655, 81.5%) to found it to be well balanced (*P*<.001), with 13.6% (89/655) finding it either easy or too easy, versus 54.7% of non–health care workers (222/406), with 39.4% (160/406) finding it either easy or too easy. Regarding length, the serious game was considered as well balanced by 89.4% of participants (health care workers: 597/655, 91.1%; non–health care workers: 351/406, 86.5%; *P*=.002).

Given the differences observed in the uptake of the French and German versions, we carried out a posthoc qualitative analysis of the final comments recorded by participants to determine whether translation problems could be involved. There were 158 comments, 81 of which (51.3%) were recorded by participants who played the German version of Escape COVID-19. Of these, 8 (9.9%) were linked with translation issues. Two examples can be found below:

The game is very good, so please refrain from using unnecessary anglicisms that are hard to understand for anyone who is not a native English speaker...

Please pay attention to correct gendering. The job title “Krankenschwester” is wrong...

All original comments are included in the curated data file (Multimedia Appendix 1).

## Discussion

### Principal Results

This nationwide campaign based on an innovative educational tool shows that giving players the impression of having an important role in the fight against the pandemic is a potent motivator for enhancing intentions of changing infection prevention and control behavior. Future serious games could use this element to promote vaccination.

Slightly less than half (509/1104, 46.1%) of participants who provided information on their intentions responded that they intended to change their infection prevention and control behavior after playing Escape COVID-19. While this figure might seem rather low at first glance, the vast majority of those not willing to change behavior answered that they already apply the guidelines outlined in the serious game. Even more reassuringly, the proportion of participants who did not believe infection prevention and control measures to be useful was extremely low. These findings must, however, be mitigated by a probably important selection bias, which is further detailed below.

The only element associated with a lesser probability of answering the second questionnaire for non–health care worker participants was choosing to play the full rather than the abridged version of the serious game. Two hypotheses might explain this finding. First, the full version of the game is longer and is much more difficult because it includes the fourth level (the ward), and non–health care workers are neither used to this setting nor to the specific personal protective equipment items and procedures depicted there. Some participants might, therefore, have chosen to abandon the game without completing this level. As the serious game was not embedded in a learning management system, we were unable to define the exact moment at which participants chose to drop out, and we were unable to gather information related to specific questions or to record the final score obtained by participants. Second, some of the participants who completed this full version of the game might have felt ill at ease completing the second questionnaire and might have thought that only health care workers were actually qualified to answer it. Nevertheless, findings from a recent study [42] strengthen support for the importance of including non–health care workers in the target population. Indeed, a secondary analysis of quantitative data collected in Ghana showed that nonclinical staff, midwives and pharmacists demonstrated lower adherence to infection prevention and control guidelines than that demonstrated by other health care workers [42]. Thus, designing infection prevention and control promotion materials that can reach many different professions, including non–health care workers in regular contact with vulnerable populations, is essential to limit viral transmission.

Older participants reported that they were more likely to change their infection prevention and control behavior after following the serious game. Three theories could explain this association. First, older age is associated with an increased risk of complications after SARS-CoV-2 infection [43], and older individuals might therefore be wary of such complications. Second, regular information material such as flyers, posters, or other text documents might be too dull and too hard to grasp [44]. A more engaging and unambiguous way of providing information might, therefore, be more appropriate to convey critical messages to this population. Indeed, even though there might be a tendency to think that games are only for young individuals, factual data bely this somewhat prejudiced impression [45-48]. Third, an effect that is linked to different digital generations (ie, X, Y and Z) could also be present. Indeed, entering an e-learning process has been found to be linked to the strongest level of contextualization of learned web-based content in generation X (individuals born between 1965 and 1979) [49].

As a federal and multilinguistic country, Switzerland represents a unique challenge. In this nationwide study, the probability of gathering health care worker data regarding the intention of changing infection prevention and control behavior and concerning perceptions about the serious game depended on language, gender, and COVID status. This last fact is hardly surprising, as it can be hypothesized that participants unwilling to detail their COVID status before playing the game might already have been skeptical regarding its content. Indeed, it does not seem unrealistic to assume that these participants did not trust the investigators and the data protection policy displayed on the website or that they were simply unwilling to provide any useful data. Though not conclusive, an element supporting this hypothesis was found in a randomized controlled trial [50] that assessed the effect of an e-learning module on attitude and knowledge regarding personal protective equipment, in which an inconsistent answer set had to be excluded and was the only one in which the respondent answered that they were unwilling to disclose their COVID status.

This study was carried out after the peak of the second pandemic wave had been reached in Switzerland, and most health care workers had already accessed a wide array of infection prevention and control educational tools, thereby substantially decreasing the number of potentially naïve participants. While gender has already been shown to influence engagement in video game activities [51], the impact of language on messages conveyed by serious game has scarcely been studied and deserves attention. Indeed, apart from the lesser probability of answering the second questionnaire, playing the game in a specific language was associated with a different probability of changing infection prevention and control behavior. One aspect of this issue is that the German and Italian translations were performed by third parties who did not take into account the multidisciplinary aspect which had driven the creation of the serious game [24]. Taking learners' profiles into account is, however, essential [52] and might have enhanced message uptake, and one participant commented on the fact that an outdated word was used to identify nurses (ie, *Krankenschwester* rather than *Pflegefachperson*).

Many studies [53,54] have already explored the adherence of particular subsets of health care workers to specific COVID-19 transmission-reducing behaviors. Recently, a web-based survey carried out in Saudi Arabia showed that respiratory therapists were more likely to adhere to infection prevention and control guidelines at home than at work [53]. Thus, behavioral change should be promoted not only in the professional setting but also in the private sphere, and motivation appears to be a key factor to promote such change. Strengthening this message even further, a systematic review [54] assessed the impact of interventions aimed at promoting eHealth capability or motivation among health care professionals, reported that most interventions aimed to promote capability rather than increasing motivation, and concluded that evidence-based developments should be carried out to enhance this last factor. Escape COVID-19, which was generated by virtue of a theory-driven process, seems to provide adequate messages, if our results are to be trusted. In addition, this serious game also helps health care workers reflect on their infection prevention and control behavior outside of the hospital environment through its first 2 levels (at home and on the road).

Cultural differences and perceptions regarding serious games, their graphics, player and nonplayer characters, and the game environment might also affect the impact of serious games. In this study, the participants who followed the German version of Escape COVID-19 were less likely to find it engaging. The reasons underlying this difference could not be thoroughly assessed, and further data should be gathered to determine how this version should be modified to increase engagement. This is critical, as the probability of actually carrying out an action relies upon this parameter [55]. In any case, including native speakers and ensuring the quality and validity of the translation is essential.

In addition, cultural and regional differences can alter whether a specific material should be considered a game, and the boundaries between serious games and gamification are often unclear [56]. However, using McCallum's [57] definition, there is little doubt that Escape COVID-19 is indeed a serious game as it is the result of the “creation of a whole new experience to achieve some change in the player.”

Obtaining a course completion certificate might have convinced some participants to complete the second questionnaire, even though more than half of those who completed it chose not to generate a certificate. Many reasons could explain this fact. First, in Switzerland, some health care workers are not required to obtain continuing medical education credits. This decreases the potential impact of the incentive of obtaining a course completion certificate. Second, official federal organizations are less used to issuing continuing medical education credits for web-based courses, particularly serious games. This prevented us from granting official continuing medical education credits to participants, which further decreased the value of this particular incentive.

### Limitations

The main limitation of this study was the use of convenience sampling, which led to a 2-tiered selection bias. First, it is to be expected that the participants who decided to register to play the game were already interested in COVID-19 protective procedures and were probably motivated to enhance their infection prevention and control behavior. Second, even after registering and playing the game, the only incentive to complete the second questionnaire was the possibility of acquiring a course completion certificate. This incentive was rather limited, as this certificate did not grant any continuing medical education credits, and many Swiss health care workers are not required to attend continuous education. In addition, this study took place after the peak of the second pandemic wave in Switzerland, which potentially decreased the interest some might have had in playing such a game. Furthermore, we were not able to determine the reasons that prevented a rather high proportion of participants from completing the second questionnaire. Attrition is to be expected in such studies [58,59], and we, therefore, strove to keep all questionnaires as short and straightforward as possible [60]. Despite our efforts, the second questionnaire might, nevertheless, have been considered too long by participants who dropped out. It is also possible that some participants who dropped out found the game ill-suited to their particular situation, unnecessary, or even boring. The high level of satisfaction among respondents is, however, relatively reassuring in this matter.

As an abridged version of the game had not been considered during the initial development process and because it was urgently requested, considerations specific to non–health care workers were neither sought nor taken into account. Therefore, even though infection prevention and control procedures are simpler and easier to follow for non–health care workers, this lack of customization might explain, at least in part, the lower willingness to change infection prevention and control behavior observed in non-health care workers. Rather than modifying Escape COVID-19 even further, the use of other serious games, such as “COVID-19-Did You Know?,” which was designed to target non–health care workers from inception, should be considered [61].

### Perspectives

The cultural differences in message uptake identified in this study are intriguing. Even though such differences have already been identified [62], the extent of these differences was quite surprising, and their causes should now be studied in order to be able to adapt future serious games to these cultural characteristics. While the original, French version of Escape COVID-19 was developed taking feedback from potential end users into account [24], the translated versions were not. Therefore, in line with the co.LAB framework, which recommends identifying learners’ profiles and adapting the game accordingly [52], we are considering carrying out focus groups to determine the shortcomings of the current version of this game and the elements that could make Escape COVID-19 more engaging. Different focus groups would be required to take into consideration elements specific to particular social, cultural, and linguistic backgrounds. In addition, such focus groups could be used to determine whether a new level or a new module could help promote vaccination. The main issues such a development must address should be explored during these sessions, which might also shed light on cultural differences regarding vaccine hesitancy [1,2,63,64].

### Conclusion

Despite the incentive of obtaining a course completion certificate, the postgame questionnaire was completed by less than half of all participants. Even though more than half of those who filled in this questionnaire reported that they were not going to change their infection prevention and control behavior, they attributed this answer to the fact that they already apply the infection prevention and control guidelines presented in the serious game. The impact of Escape COVID-19 on participants who intended to change their infection prevention and control behavior after playing was, however, substantial as these users were generally radical in their willingness to alter their practice. They reported that their willingness to change their infection prevention and control behavior was equally motivated by the feeling of playing an important role in the common effort against the pandemic and by the information contained in the serious game.

In this study, older age was associated with the intention of changing infection prevention and control behavior. Conversely, playing the German version of the game decreased the likelihood of intending to change behavior. Different hypotheses could explain these findings and should now be explored to help adapt the game and enhance the uptake of infection prevention and control messages.

## Appendix

Multimedia Appendix 1 Curated data file in CSV and DTA formats.
